# Development of polymyxin- and aminoglycoside-based outer membrane permeabilizers: a review

**DOI:** 10.3389/fmicb.2025.1625300

**Published:** 2025-09-18

**Authors:** Danzel Marie Ramirez, Frank Schweizer

**Affiliations:** ^1^Department of Chemistry, University of Manitoba, Winnipeg, MB, Canada; ^2^Department of Medical Microbiology and Infectious Diseases, University of Manitoba, Winnipeg, MB, Canada

**Keywords:** polymyxins, aminoglycosides, outer membrane permeabilizers, combination therapy, antibiotic adjuvants, potentiators

## Abstract

The prevalence of antimicrobial resistance (AMR) necessitates the development of alternative therapeutic options, particularly against critical priority Gram-negative pathogens. The utilization of antibiotic adjuvants or potentiators is an advantageous strategy that targets bacterial resistance mechanisms, thereby augmenting the activity of an antibiotic used in combination. Among these, outer membrane (OM) permeabilizers are a promising class of adjuvants which compromise the OM barrier unique to Gram-negative bacteria. This review focuses on the emerging role of polymyxins and aminoglycosides – two structurally distinct antibiotics with different modes of action, but share the ability to interact with the bacterial OM. Here, we explore the design, modification, and application of polymyxin- and aminoglycoside-based OM permeabilizers, highlighting their potential against resistant Gram-negative infections.

## 1 Introduction

### 1.1 Antibiotic resistance

Antibiotic resistance is a natural process (D’Costa et al., 2011) and environmental bacteria possess ancient intrinsic resistance mechanisms predating the introduction of antibiotics in the clinic (Cox and Wright, 2013). However, the continuous application of antibiotics has contributed greatly to the selective pressure that promoted the dissemination of other resistance determinants (Davies and Davies, 2010; Perry et al., 2016). The prevalence of antimicrobial resistance (AMR) is now a global health threat (Murray et al., 2022). In 2021, it was estimated that 1.27 million deaths were directly attributable to bacterial AMR, while 4.71 million deaths were associated with AMR (Naghavi et al., 2024). A statistical model forecasted that the AMR burden will continue to increase to 1.91 million attributable and 8.22 million associated mortalities in 2050 (Naghavi et al., 2024).

AMR is multifaceted and tackling this problem involves a tailored approach for different regions (Murray et al., 2022). For instance, in low- and middle- income countries (LMICs) where the burden is high and first-line antibiotics fail, ensuring the availability and accessibility to second-line treatment options is required (Laxminarayan et al., 2013; Murray et al., 2022). Conversely, antibiotic stewardship is more beneficial for countries wherein the overuse and misuse of antibiotics are the main drivers of AMR (Murray et al., 2022). Other issues such as inadequate healthcare infrastructure, trained personnel, and antibiotic surveillance systems also need to be addressed, particularly in LMICs with weak health systems (Laxminarayan et al., 2013). It was forecasted that if the healthcare quality for infectious diseases and access to antibiotics were improved, the number of cumulative deaths that could be prevented between 2025 and 2050 was 92.0 million (Naghavi et al., 2024). More importantly, there is an urgent need for the innovation of novel antibiotics, as AMR threatens the effectiveness of existing treatments. Under a scenario wherein an anti-Gram-negative antimicrobial is successfully developed, an estimated 1.1 million AMR deaths could be avoided by 2050 (Naghavi et al., 2024). In spite of this, antibiotic discovery has declined, with large pharmaceutical companies no longer investing in antibiotics due to the unprofitable market (Miethke et al., 2021). Push incentives are necessary to promote research and reduce the cost of drug development by funding different stages – from hit generation to market utilization (Cama et al., 2021; Miethke et al., 2021). Pull incentives aim to provide financial viability to raise the return on investment post-approval (Årdal et al., 2017; Bhavnani et al., 2020), particularly for narrow-spectrum antibiotics reserved for drug-resistant infections which are difficult to commercialize (Wells et al., 2024). In 2023, there were 244 potential candidates in the preclinical pipeline (Gigante et al., 2024) and 97 products in the clinical pipeline (Melchiorri et al., 2024). However, the current pipeline and recently approved antibiotics were deemed insufficient by the World Health Organization (WHO) in addressing the accelerating emergence of AMR (Prasad et al., 2022; Melchiorri et al., 2024). One of the bases in the evaluation is adhering to the bacterial priority pathogens list (Melchiorri et al., 2024), which serves as a guideline for antibiotic research and development (World Health Organization, 2024). Gram-negative bacteria resistant to last-resort antibiotics dominate the list, with carbapenem-resistant *Acinetobacter baumannii*, *Enterobacterales*, and *Pseudomonas aeruginosa* among the critical and high priority pathogens (World Health Organization, 2024). While Gram-negative pathogens notably maintain their top ranking in the published list (World Health Organization, 2024), majority of the antibiotics in our current arsenal are only effective against Gram-positive bacteria (Saxena et al., 2023). Overall, these issues highlight an urgent gap in infectious disease therapy, and it is crucial to explore effective treatment options against Gram-negative pathogens.

### 1.2 The outer membrane of Gram-negative bacteria is a permeability barrier

**Figure 1:**
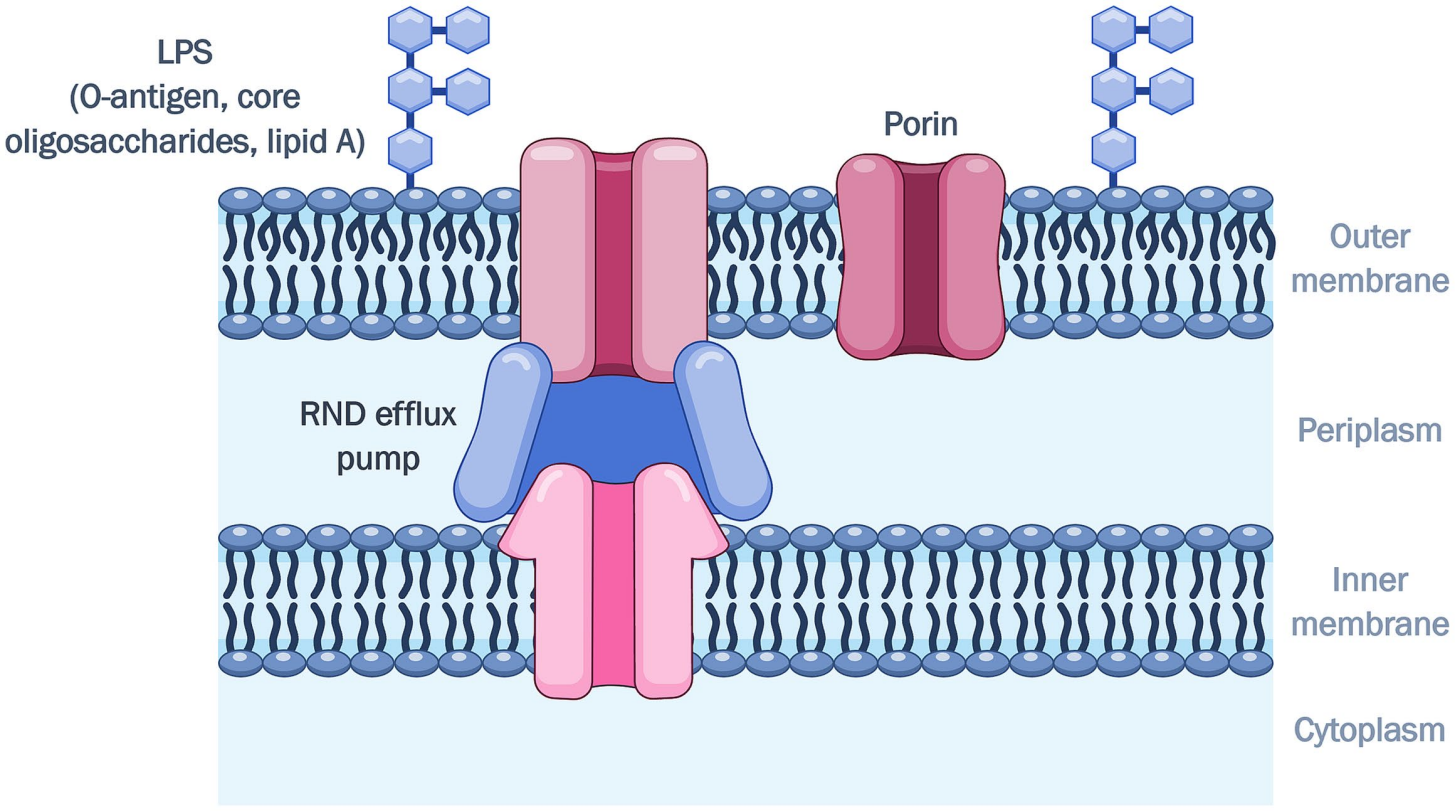
Outer membrane of Gram-negative bacteria.

Gram-negative bacteria are notoriously more difficult to treat, as they possess an outer membrane (OM) in addition to the cytoplasmic or inner membrane, which serves as an intrinsic resistance mechanism that reduces drug uptake (Reygaert, 2018). The decreased influx of drugs also works synergistically with resistance-nodulation-cell division (RND)-type efflux pumps, which span both membranes and actively extrude antibiotics (Nikaido and Takatsuka, 2009). Since the OM is the interface between the bacterial cell and the external environment, the first challenge for a drug molecule is to overcome this permeability barrier. This asymmetric lipid bilayer is comprised of lipopolysaccharides (LPS) on the outer leaflet, phospholipids in the inner leaflet, and various OM proteins (OMPs) (Delcour, 2009; Figure 1).

The LPS consists of the O-antigen, core oligosaccharides, and lipid A. The O-antigen polysaccharides are the exposed portion of the LPS, extending out into the environment (Lerouge and Vanderleyden, 2002; Kim et al., 2016). The O-antigen is the hydrophilic domain of the LPS, with oligosaccharides that vary in chain lengths and sugar content, giving rise to differences in the antigenic typing between Gram-negative species and strains (Lerouge and Vanderleyden, 2002). The presence of the O-antigen renders the LPS “smooth,” while a truncated LPS lacking the O-antigen or lipooligosaccharides (LOS), are termed “rough” (Lerouge and Vanderleyden, 2002; Raetz and Whitfield, 2002). The core oligosaccharides consist of variable sugar residues in the outer region, and a well-preserved inner region consisting of 3-deoxy-D-manno-oct-2-ulosonic acid (Kdo) and L-glycero-D-mannose-heptopyranose (Hep) (Frirdich and Whitfield, 2005). Two Kdo units are linked to lipid A (Delcour, 2009), and these two components which form the Kdo_2_-lipid A complex are the minimum requirement for growth in Gram-negative bacteria (Raetz et al., 2007). The Hep phosphates bind strongly with divalent cations which bridge LPS molecules, and these interactions provide structural integrity to the OM (Frirdich and Whitfield, 2005). It is evident that the conservation of the inner core plays a crucial role in OM permeability (Frirdich and Whitfield, 2005), as mutant Hep-deficient “deep rough” strains have a more penetrable OM (DeLucia et al., 2011). The innermost component is lipid A, the hydrophobic portion of the LPS which anchors it to the OM (Garcia-Vello et al., 2022). The lipid A is a glucosamine disaccharide acylated with 3-hydroxymyristic acid (Delcour, 2009). The presence of additional saturated fatty acid chains resulting in tight packing, are attributed to the increased hydrophobicity of the asymmetric bilayer relative to a phospholipid bilayer (Vaara and Nurminen, 1999; Delcour, 2009). The glucosamine backbone is also phosphorylated and bridged via electrostatic interactions with divalent magnesium or calcium cations (Snyder and McIntosh, 2000; Delcour, 2009; Ude et al., 2021; Sun et al., 2022) which contribute to the barrier function of the OM (Vaara and Nurminen, 1999).

The most abundant OMPs in Gram-negative bacteria are porins (Choi and Lee, 2019), which form water-filled pores that facilitate passive diffusion of small, hydrophilic molecules (Choi and Lee, 2019). These channels can be non-specific or substrate-specific. The large, general diffusion porins in *Enterobacterales* allow entry of molecules with masses up to 600 Da, while the specific porins in *P. aeruginosa* and *A. baumannii* have an exclusion limit of 200 Da (Ude et al., 2021). The absence of non-specific porins OmpF and OmpC in *P. aeruginosa* and *A. baumannii* (Chevalier et al., 2017) makes the OM of these organisms less permeable in comparison to *E. coli* (Ude et al., 2021), which has an abundance of these porins. Furthermore, porins such as OmpA and OprF are involved in maintaining membrane integrity by stabilizing links between the OM and the peptidoglycan (Choi and Lee, 2019; Ude et al., 2021).

Altogether, the various components of the OM contribute to forming an effective permeability barrier. The preservation of the inner core, rigidity of the lipid interior of the LPS, and presence of certain porins provide stability to the OM (Vaara and Nurminen, 1999; Delcour, 2009; Bertani and Ruiz, 2018). The hydrophilic character of the O-antigen and core region, as well as porins, impart low permeation for hydrophobic molecules (Hiroshi, 2003; Delcour, 2009; Fernández and Hancock, 2012; Bertani and Ruiz, 2018). Porins also have a size exclusion for hydrophilic molecules (Fernández and Hancock, 2012). Therefore, antibiotics with these physicochemical properties diffuse slowly across the OM and have limited activity against Gram-negative bacteria. In addition, the development of adaptive resistance can cause mutations resulting in low expression, loss or alterations of porins (Fernández and Hancock, 2012), thereby conferring resistance to porin-mediated antibiotics such as *β*-lactams and fluroquinolones (Hancock and Bell, 1988; Hiroshi, 2003).

## 2 Outer membrane permeabilizers enhance antibiotic uptake

OM permeabilizers are a class of antibiotic adjuvants, potentiators, or resistance breakers (Melander and Melander, 2017; Douafer et al., 2019; Laws et al., 2019), which target the inherent resistance mechanism of OM impermeability in Gram-negative bacteria (Vaara, 1992). These agents interact with the lipid A component of the LPS leaflet, whereby the cationic groups of the OM perturbant form electrostatic interactions with the negatively charged phosphates (Vaara, 1992). This displaces stabilizing divalent magnesium or calcium cations, initially intercalated between phosphate groups, leading to increased OM permeability (Vaara, 1992). In addition, the hydrophobic portion of an OM permeabilizer can insert within the fatty acyl chains in lipid A, resulting in further membrane expansion (Velkov et al., 2010). These combined effects disrupt the OM and consequently improve the periplasmic and/or intracellular concentration of a partner antibiotic.

The potentiation of OM impermeable antibacterial agents is of particular interest as they will greatly benefit from enhanced OM permeation. Moreover, being able to extend the activity spectrum toward Gram-negative bacteria is imperative, given the limited treatment options and their designation as high-priority pathogens by the WHO due to rising multidrug resistance. OM permeabilizing adjuvants have also been shown to enhance to activity of agents not necessarily hindered by the OM, and in the presence of additional resistance mechanisms such as inactivating enzymes and efflux (Melander and Melander, 2017). Thus, porin-mediated *β*-lactams and β-lactamase inhibitors (BLIs) present themselves as suitable partner antibiotics. They have more accessible periplasmic targets, and there is substantial evidence, particularly from their synergy with aminoglycosides, that suggest they benefit from improved OM permeability. Potentiating β-lactam/β-lactamase inhibitor (BL/BLI) combinations is also important given their widespread clinical use. The efflux-susceptible tetracyclines are also notable candidates, as their initial bacteriostatic or growth inhibitory effects can be enhanced to bactericidal action when combined with an OM perturbant (Qu et al., 2019). Moreover, the synergistic interaction is characterized by enhanced activity with a reduction in the concentration of both agents, thereby lowering the required dose which can alleviate toxic effects (Melander and Melander, 2017). This strategy also reduces the emergence of resistance, as OM permeabilizers do not possess standalone activity (Melander and Melander, 2017). Among the most notable OM permeabilizers are polymyxins, aminoglycosides, and their analogs, which will be discussed in detail in this review.

### 2.1 Polymyxins and their analogs as outer membrane permeabilizers

Polymyxins are antimicrobial peptides isolated from *Bacillus polymyxa*, which exert their bactericidal effect by targeting the bacterial OM (Velkov et al., 2010). In probing the precise mechanism by which polymyxins bind to LPS, atomic force microscopy (AFM) experiments have elucidated that polymyxins form hexagonal crystalline structures with LPS on the surface of OM patches of *E. coli* (Manioglu et al., 2022). Based on surface plasmon resonance studies, it has also been proposed that polymyxins initially bind transiently with LPS, followed by membrane insertion as nucleates. These species then self-associate and accumulate as stable, long-lived clusters (Buchholz et al., 2024). Polymyxins are then presumed to disrupt the physical integrity of the phospholipid bilayer of the inner membrane (Velkov et al., 2010). The polymyxins straddle the interface of the hydrophilic head groups and fatty acyl chains, resulting in membrane thinning (Velkov et al., 2010). This causes cell lysis and leakage of cytoplasmic content (Poirel et al., 2017). However, there is evidence that suggests polymyxins target LPS present in the inner membrane (Sabnis et al., 2021).

**Figure 2:**
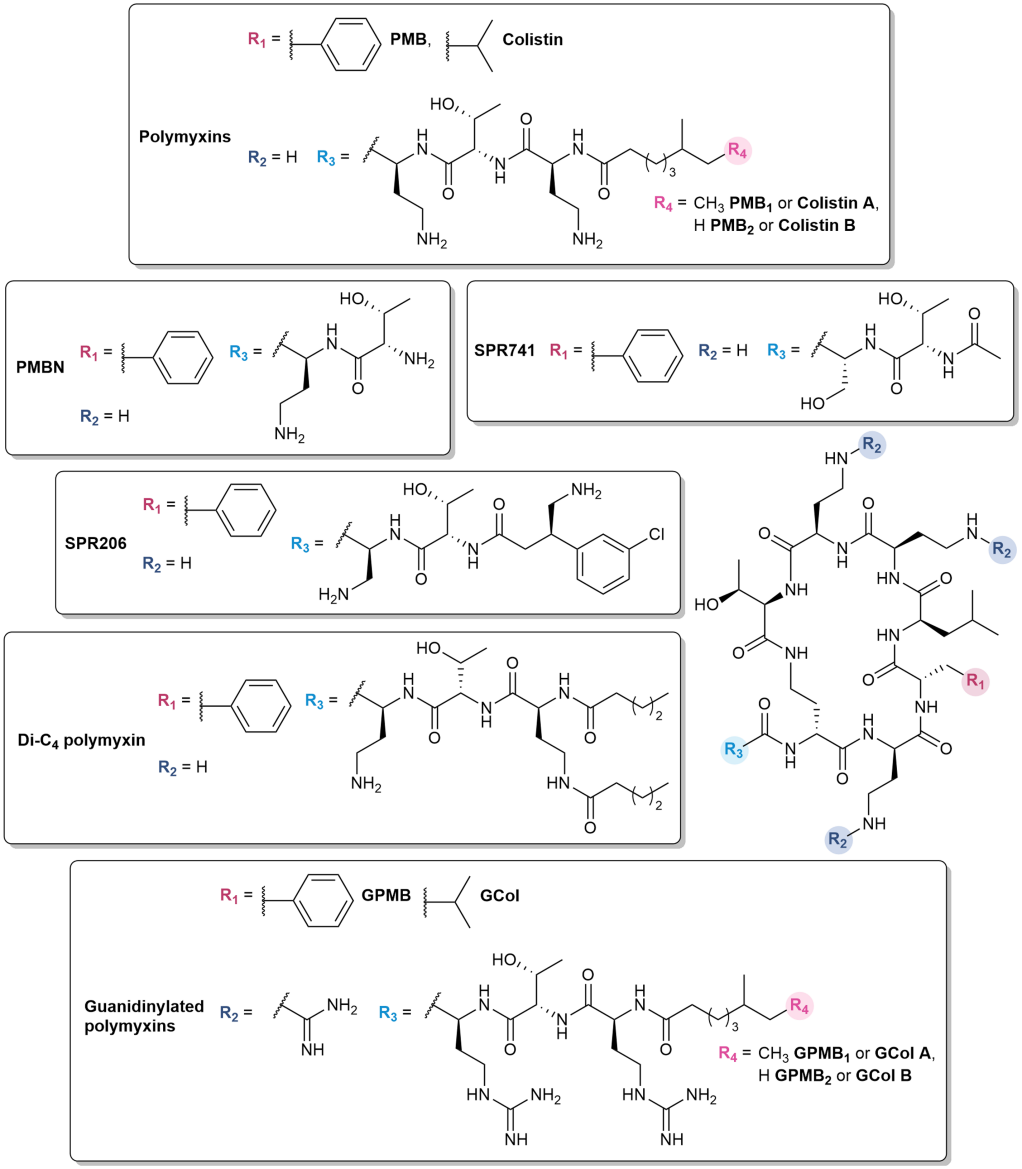
Structures of polymyxins and their derivatives.

Despite their effectiveness, the associated neurotoxicity (Poirel et al., 2017) and nephrotoxicity (Gai et al., 2019) has restricted their clinical application as last-resort antibiotics for infections caused by multidrug-resistant (MDR) Gram-negative pathogens (Phe et al., 2014). The polymyxins used in clinical practice are polymyxin E (colistin) and B (PMB), which differ by a D-leucine and D-phenylalanine, respectively, at position 6 within the peptide ring, (Figure 2). Moreover, these polymyxins are a mixture of polypeptides (Moubareck, 2020). Colistin is composed of colistin A and B, while the major components of PMB are PMB_1_ and PMB_2_, which differ by a single methyl group in the lipid portion (Figure 2).

Beyond their direct antibacterial activity, polymyxins have been repurposed and widely reported to synergize with aminoglycosides, *β*-lactams, BL/BLI combinations, fluoroquinolones, tetracyclines, macrolides, glycopeptides, lipoglycopeptides, fosfomycin, rifampicin, daptomycin, and chloramphenicol against Gram-negative bacteria (Elemam et al., 2010; MacNair et al., 2018; Lin et al., 2019; Shinohara et al., 2019; Abdul Rahim et al., 2021; Ontong et al., 2021; Wickremasinghe et al., 2021; Ju et al., 2022; Mantzana et al., 2023; Nwabor et al., 2023; Olsson et al., 2024; Wang et al., 2024b; Wang et al., 2024a; Van Den Berg et al., 2025). While it is established that polymyxins augment the activity of a partner antibiotic by disrupting the OM, the synergistic effect could also arise from the complementary mechanisms of both agents. A study demonstrated that novobiocin was able to stimulate LPS transport by binding to the LptB ATPase, thereby enhancing the activity of polymyxins (Mandler et al., 2018).

Polymyxin analogs have also demonstrated the ability to potentiate different antibiotics. In essence, some of the most effective known OM permeabilizers are derived from polymyxins – the “gold standard” polymyxin B nonapeptide (PMBN) (Vaara and Vaara, 1983) and SPR741 (formerly NAB741) (Vaara et al., 2010) which completed phase 1 clinical trials (Eckburg et al., 2019). Due to the toxic nature of polymyxins, emphasis on alleviating nephrotoxicity (Danner et al., 1989; Vaara, 1992; Vaara et al., 2010; Nilsson et al., 2015; Corbett et al., 2017; Zurawski et al., 2017) was factored in the design of these polymyxin-based OM permeabilizers, in which the key modifications involve the removal of the *N*-terminal lipid tail and reducing the number of 2,4-diaminobutyric (Dab) residues.

#### 2.1.1 Deacylated polymyxins retain OM permeabilizing capabilities

Polymyxins with truncated fatty acyl chains are weakly or non-bactericidal due to the absence of the lipid tail which is crucial for eliciting the lethal action of polymyxins (Vaara, 1992). Nonetheless, polymyxin decapeptides (deacylated polymyxin (DAPB) and colistin), PMB and colistin nonapeptides, and PMB octapeptide and heptapeptide demonstrated the ability to permeabilize the OM (Kimura et al., 1992; Vaara, 1992). Among these analogs, PMBN (Figure 2) was the most effective OM permeabilizer and retains the ability to bind to the LPS (Tsubery et al., 2000; Moison et al., 2017). It synergizes *in vitro* with antibiotics such as rifampicin, novobiocin, and erythromycin against *E. coli*, *K. pneumoniae*, and *P. aeruginosa* (Vaara, 2019). It also demonstrates *in vivo* efficacy in combination with novobiocin and erythromycin, and protected mice infected with *K. pneumoniae* and *P. aeruginosa* (Ofek et al., 1994). While PMBN was less toxic than polymyxins in several animal studies, it was found that it was still as nephrotoxic as PMB, and was therefore no longer considered for clinical use (Vaara, 1992, 2010). PMBN is still contemporarily used as a tool for the development of OM permeabilizers, evident in several studies (Vaara, 2019). It has been widely investigated since its discovery, and its well-characterized properties have established PMBN as the benchmark for evaluating novel OM permeabilizers (Vaara and Vaara, 2010; Vaara, 2019; Wesseling and Martin, 2022).

#### 2.1.2 Polymyxins with fewer charges show reduced nephrotoxicity

The nephrotoxicity of PMBN prompted the development of polymyxins with three positive charges. NAB7061 possessed variations in the linear peptide segment with an octanoyl as the fatty acid tail, the *C*-terminal Dab replaced with an aminobutyryl residue, and absence of the *N*-terminal Dab (Vaara et al., 2008). NAB7061 was notably synergistic with rifampin, clarithromycin, azithromycin, erythromycin, and mupirocin against *E. coli*, *K. pneumoniae*, and *Enterobacter cloacae* (Vaara et al., 2008; Vaara et al., 2010). Rifampicin and clarithromycin potentiation was also observed in *A. baumannii* (Vaara et al., 2008). The *in vivo* potency of the combination therapy of NAB7061 and erythromycin was demonstrated in an experimental *E. coli* murine peritonitis model (Vingsbo Lundberg et al., 2010).

Further optimization led to the development of SPR741, with the *N*-terminal lipid tail substituted with an acetyl group and one of the Dab residues replaced with serine (Vaara et al., 2010; Figure 2). SPR741 synergized with multiple antibiotics such as rifampicin (Corbett et al., 2017; Zurawski et al., 2017; Vaara, 2019), clarithromycin (Corbett et al., 2017; Vaara, 2019), erythromycin (Corbett et al., 2017), azithromycin (Corbett et al., 2017; Stainton et al., 2018), ceftazidime (Eckburg et al., 2019), and piperacillin-tazobactam (Eckburg et al., 2019) against a variety of Gram-negative bacteria. The phase 1 studies of SPR741 demonstrated its safety and pharmacokinetics in combination with ceftazidime, aztreonam, and piperacillin-tazobactam (Eckburg et al., 2019). However, further development of SPR471 was discontinued in favor of the standalone antibacterial agent SPR206 (Figure 2), a potent, non-nephrotoxic PMB derivative which may offer improved safety and efficacy profiles (Prasad et al., 2022). It demonstrated both in vitro activity and in vivo efficacy against MDR Gram-negative bacteria (Brown et al., 2019; Bruss et al., 2021, 2023). In comparison to SPR741, SPR206 bears an aminobutyrate N-terminus with a 3-chlorophenyl moiety at the *β*-position (Brown et al., 2019).

#### 2.1.3 Dilipid polymyxins modulate membrane selectivity

Dilipid polymyxins were synthesized with the hypothesis that additional hydrophobic character in the lipid component would enhance its ability to insert into membranes (Domalaon et al., 2018a). The Dab amine side chain was acylated with various hydrophobic components: butyl, octyl, and dodecyl lipids, as well as adamantyl and biphenyl groups. Among these series of compounds, the butyric acid derivative (Figure 2) potentiated β-lactams, tetracyclines, fluoroquinolones, fosfomycin, trimethoprim, chloramphenicol, novobiocin, vancomycin, clindamycin, linezolid, and rifampicin against *P. aeruginosa* PAO1 in a manner comparable to PMBN (Domalaon et al., 2018a). While the dilipid polymyxins did not necessarily improve the activity against Gram-negative bacteria, it conferred anti-Gram-positive activity, implying that incorporation of another lipid potentially strengthens its interactions with the lipoteichoic acid in Gram-positive bacteria via hydrophobic effects. The increased hydrophobicity could also result in non-specific lysis of eukaryotic cells. A preliminary assessment showed that the dilipid butyric acid derivative was non-hemolytic at 512 μg/mL similar to colistin and PMBN (Domalaon et al., 2018a).

#### 2.1.4 Guanidinylated polymyxins enhance outer membrane permeabilization

Substitution of the Dab amines to guanidines was postulated to favor interactions with the OM (Kim et al., 2021). In comparison to amines, guanidinium groups have a higher p*K*_a_ and remain protonated across a wide pH range, including physiological pH (Blondeau et al., 2007; Kim et al., 2021). The delocalization of the positive charge and planar Y-shape geometry allow them to bind with high affinity to oxoanions (Blondeau et al., 2007), such as the phosphates on the core sugars and lipid A of the LPS (DeLucia et al., 2011). In a recent study, cationic peptide-based adjuvants with guanidine-containing arginine residues showed greater antibiotic potentiation compared to those with lysine and Dab (Ramirez et al., 2020). The resulting guanidinylated colistin (GCol) and PMB (GPMB) (Figure 2) synergized with a panel of antibiotics against reference and MDR strains of Gram-negative bacteria (Ramirez et al., 2022). The activity spectrum of rifampicin and erythromycin were expanded to Gram-negative bacteria, with minimum inhibitory concentrations (MICs) below the interpretative susceptibility breakpoint against MDR clinical isolates (Ramirez et al., 2022). In particular, rifampicin potentiation by the guanidinylated polymyxins was higher than PMBN in colistin-resistant *P. aeruginosa*, *E. coli*, *K. pneumoniae*, and *E. cloacae* (Ramirez et al., 2022). The guanidinylated polymyxins also enhanced the activity of ceftazidime and aztreonam, and their respective combinations with the BLI avibactam against MDR and *β*-lactamase harboring *P. aeruginosa*, respectively (Ramirez et al., 2022). Furthermore, the triple combinations of guanidinylated colistin with ceftazidime/avibactam or aztreonam/avibactam were shown to be bactericidal (Ramirez et al., 2022). Despite the lack of standalone activity, fluorescent assays measuring the uptake of the membrane impermeable probe, *N*-phenyl-1-naphthylamine (NPN), demonstrated that the guanidinylated polymyxins permeabilized the OM in a time- and concentration-dependent manner similar to PMBN (Ramirez et al., 2022). Unfortunately, changing the amines to guanidines did not improve the cytotoxicity relative to the parent PMB (Ramirez et al., 2022).

#### 2.1.5 Polymyxin hybrids synergize with conjugated drugs

**Figure 3:**
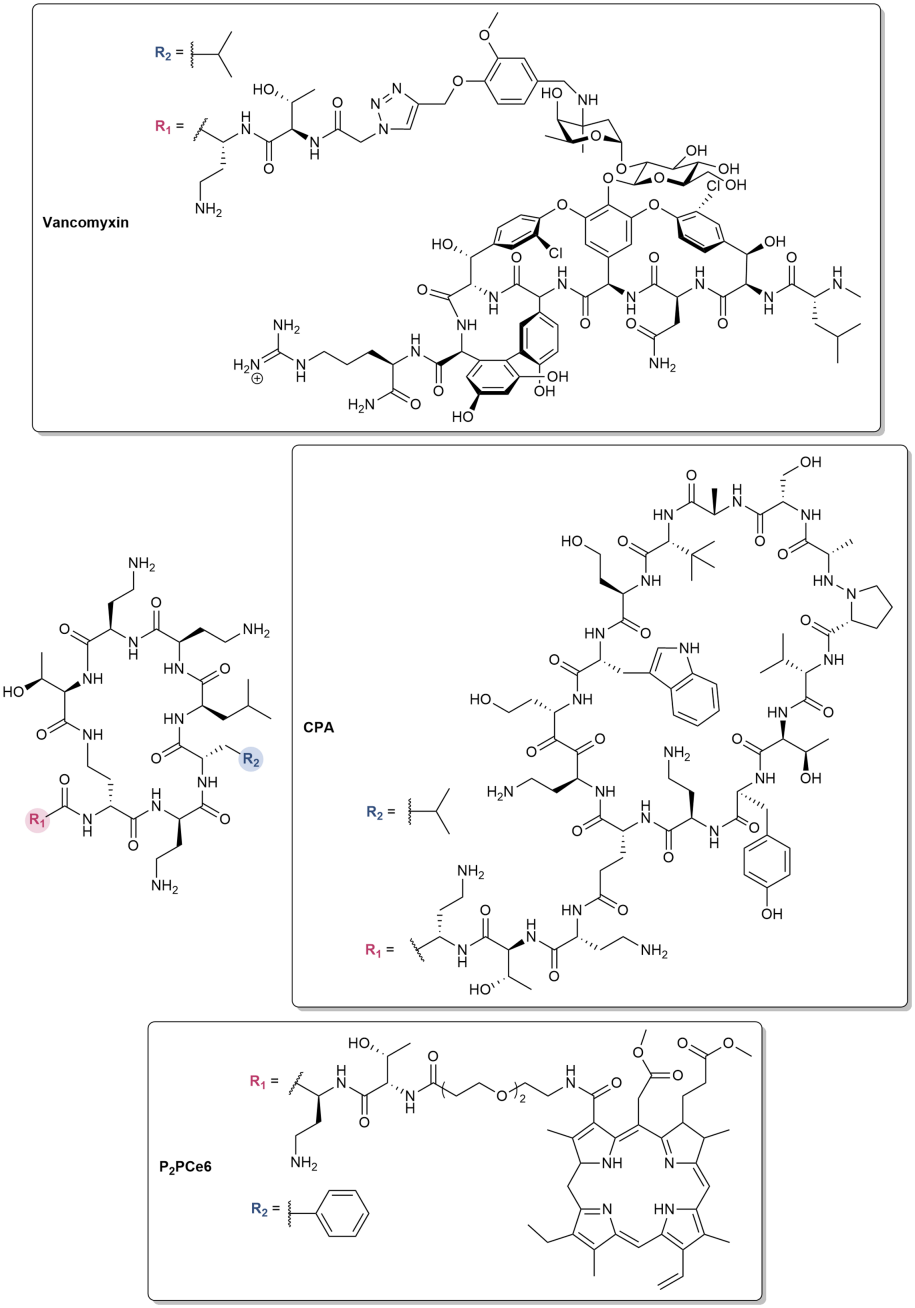
Structures of polymyxin hybrids.

Polymyxins are also utilized as pharmacophores in the design of antibiotic hybrids, wherein two or more bioactive scaffolds are covalently linked while preserving their distinct mechanisms of action, resulting in a synergistic effect (Domalaon et al., 2018b). For instance, vancomyxins (Figure 3) are created by covalently linking vancomycin to polymyxin E nonapeptide (PMEN) (Van Groesen et al., 2021). The LPS binding of the hybrids are maintained, resulting in the improved activity of the conjugated vancomycin against Gram-negative bacteria relative to the dual combination of vancomycin and PMEN (Van Groesen et al., 2021). Against Gram-positive bacteria, the potency of vancomycin is retained, with some enhancement against vancomycin-resistant strains (Van Groesen et al., 2021). Compared to clinically used polymyxins, the hybrids also showed reduced nephrotoxicity (Van Groesen et al., 2021). PMEN was conjugated to a β-hairpin peptide macrocycle inspired by murepavadin, a peptidomimetic antibiotic which inhibits the LPS transport protein LptD in *P. aeruginosa* (Luther et al., 2019). The potent activity of the resulting chimeric peptidomimetic antibiotics (CPA) (Figure 3) against MDR pathogens arises from their ability to target both the LPS and BamA (Luther et al., 2019). BamA is an OMP and a key component of the β-barrel assembly machinery (BAM), which is responsible for the folding and integration of β-barrel proteins in the OM. Aside from antibiotics, PMBN was covalently attached to the FDA-approved photosensitizer chlorin e6 (Ce6) via different linkers (Wu et al., 2024). The ability of PMBN to permeabilize the OM allowed diffusion of Ce6 into the bacterial membranes, resulting in photobactericidal activity (Wu et al., 2024). The optimized derivative possessing an amino-3,6-dioxaoctanoic linker (P2pCe6) (Figure 3) showed selective imaging of Gram-negative bacteria, improved biocompatibility, higher reactive oxygen species (ROS) production, and strong photoinactivation of Gram-negative bacteria (Wu et al., 2024). P2pCe6 was also effective in treating *P. aeruginosa* infections and accelerating wound healing in *Galleria mellonella* and mouse models, respectively (Wu et al., 2024).

#### 2.1.6 Polymyxin delivery systems for controlled drug delivery

The use of polymers, conjugates, liposomes, gels, fibers, and membranes to improve the delivery of polymyxins has been widely studied (Dubashynskaya and Skorik, 2020). These drug carriers are designed to control the release of polymyxins, ensuring targeted delivery, increased bioavailability, improved chemical and physical stability, and enhanced accumulation in membranes and biofilms (Dubashynskaya and Skorik, 2020). As a result, these systems increase the local concentration of polymyxins, allowing for reduced dosages and lower toxicity (Dubashynskaya and Skorik, 2020). A self-forming peptide-based hydrogel was utilized for the sustained delivery of PMB. The hydrogels were formed by mixing PMB with a solution of varying peptide amphiphiles with tunable mechanical properties (Shi et al., 2021). The system released the drug over 5 days, resulting in prolonged antibacterial activity against Gram-negative bacteria (Shi et al., 2021). The localized release of PMB reduced *P. aeruginosa*-related mortality in a *G. mellonella* burn wound model (Shi et al., 2021). Moreover, the inclusion of fusidic acid in the polymyxin hydrogel enhanced the activity against *S. aureus* and *A. baumannii*, highlighting the potential application of this delivery system in combination therapy (Shi et al., 2021).

PMB has also been strategically anchored to the carrier, facilitating the co-delivery of companion antibiotics (Wu et al., 2022). For instance, linezolid was encapsulated inside mesoporous silica particles, and the outer surface was coated with PMB (Otri et al., 2024). The PMB coating disrupted bacterial membranes, enhancing the penetration and efficacy of linezolid (Otri et al., 2024). The system dramatically improved activity against *E. coli*, as well as Gram-positive bacteria (Otri et al., 2024). Another study involved the development of anionic liposomes loaded with fosfomycin, with the cationic PMB adsorbed onto the surface, enabling selective bacterial targeting against *A. baumannii* (Zhu et al., 2023). *In vitro* and *in vivo* tests showed that these PMB–fosfomycin liposomes achieved stronger antibacterial and anti-inflammatory effects than free drug mixtures, while also reducing the nephrotoxicity of PMB (Zhu et al., 2023). PMB-based platforms utilizing covalent organic nanoparticles, nanocrystals, and liposomes (Le Guern et al., 2017; Jiang et al., 2021; Cui et al., 2024) have also been applied in photodynamic therapy, wherein they act synergistically with a photosensitizer.

### 2.2 Aminoglycosides and their analogs as outer membrane permeabilizers

**Figure 4:**
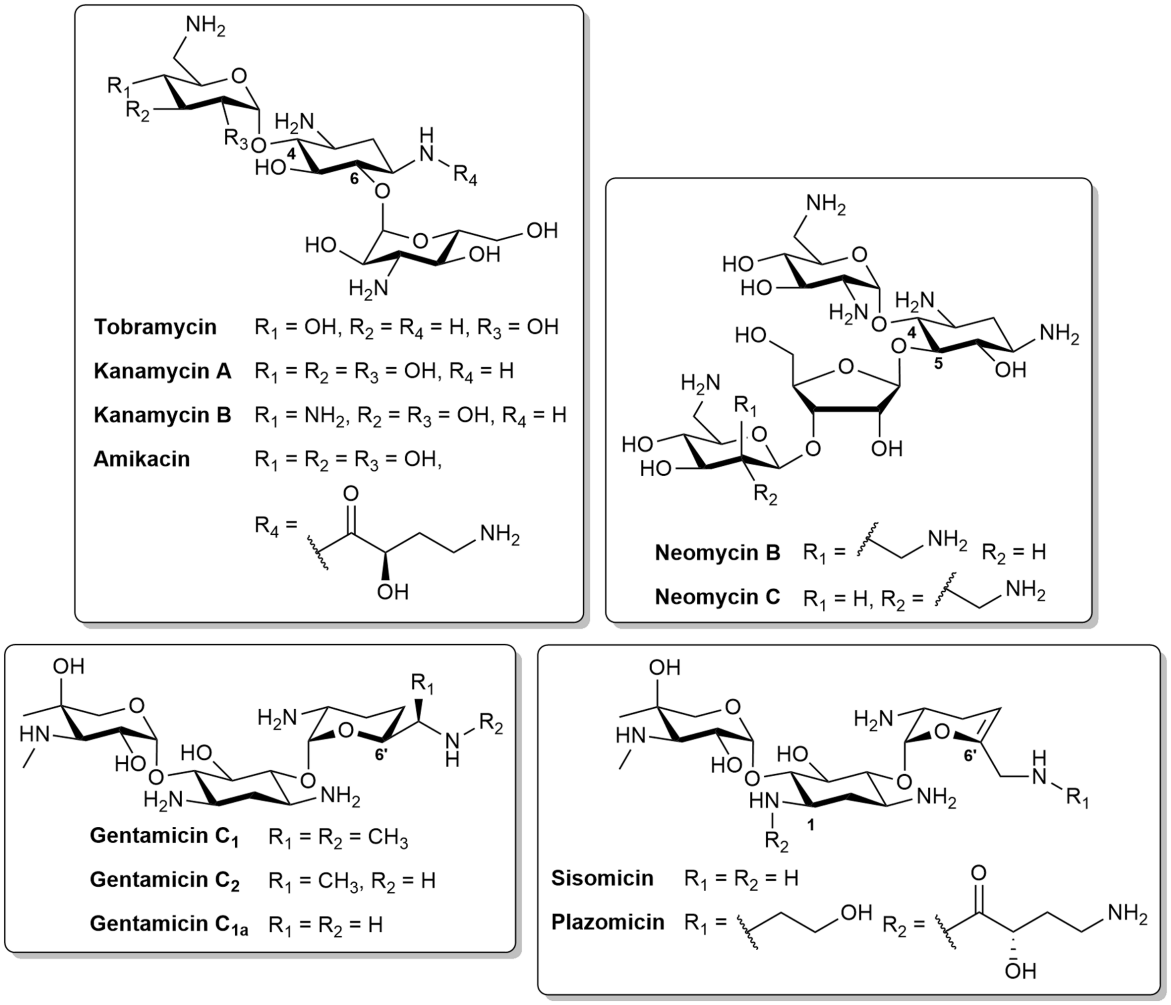
Structures of different aminoglycosides.

Aminoglycosides are a class of pseudo-oligosaccharide antibiotics capable of transporting across the OM via a “self-promoted uptake” mechanism (Krause et al., 2016). Aminoglycosides are natural products isolated from actinomycetes or semisynthetic derivatives (Serio et al., 2018). They contain a core cyclitol ring linked to several amino sugars via a glycosidic bond (Mingeot-Leclercq et al., 1999). The most common 2-deoxystreptamine ring can be further subdivided based on substitution patterns: tobramycin, kanamycin, amikacin, gentamicin, sisomicin and plazomicin are 4,6-disubstitued, whereas neomycin B is 4,5-disubsituted (Figure 4). Amikacin is an example of a semisynthetic aminoglycoside derived from kanamycin, with one of the amine functions in the cyclitol ring substituted with an L-hydroxyaminobuteroyl amide (Figure 4). Sisomicin is similar to gentamicin, but with unsaturation in ring III. The recently developed plazomicin (Aggen et al., 2010) is a semisynthetic derivative of sisomicin, with 2-hydroxyethyl and hydroxylaminobutyric acid groups at positions 1 and 6′, respectively (Figure 4). Aminoglycosides can also be a combination of different constituents. The active components of neomycin are the two stereoisomers, neomycin B and C (Figure 4). Gentamicin is composed of gentamicin C_1_, C_2_, and C_1a_ which have different methyl substitutions at position 6′ (Figure 4).

The clinical significance of aminoglycosides is marked by their broad-spectrum bactericidal activity, effective against various Gram-negative and Gram-positive bacteria, as well as mycobacteria (Serio et al., 2018). The initial uptake of aminoglycosides is then followed by entry into the cytoplasm via a rate-limiting, energy-dependent electron transport (Mingeot-Leclercq et al., 1999). In the cytosol, the aminoglycosides then bind to the A-site of the 16S ribosomal RNA (rRNA) of the 30S ribosome (Krause et al., 2016). This binding leads to the inhibition of tRNA translocation and consequently protein synthesis (Garneau-Tsodikova and Labby, 2016). Moreover, the binding affinity to tRNA increases, which allows the continuation of translation despite incorrect mRNA-tRNA pairing, resulting mistranslated proteins (Carter et al., 2000; Garneau-Tsodikova and Labby, 2016). In comparison to other aminoglycosides, tobramycin can induce cell death via two mechanisms – inhibition of protein translation at low concentrations (<4 μg/mL), or permeabilization of the OM at higher concentrations (≥8 μg/mL) (Bulitta et al., 2015).

In addition to the use of aminoglycosides in monotherapy, they have exhibited synergistic interactions with other antibiotic classes (Krause et al., 2016). In particular, the inclusion of an aminoglycoside to *β*-lactams and BL/BLI combinations has been recommended as therapy for *P. aeruginosa* infections (Bassetti et al., 2018). Gentamicin/ampicillin covers group B streptococcus and *E. coli* for treating neonatal sepsis (Simonsen et al., 2014). Amikacin has been extensively reported to synergize with various BL/BLI combinations such as piperacillin/tazobactam (Burgess and Hastings, 2000), ceftolozane/tazobactam (Noel et al., 2018; Galani et al., 2020), and imipenem/relebactam (Asempa et al., 2019). Studies have also displayed the effectiveness of tobramycin with piperacillin/tazobactam (Joshi et al., 1999) and *in vitro* synergy with ceftazidime/avibactam (Mataraci Kara et al., 2020).

#### 2.2.1 Non-ribosomal tobramycin conjugates synergize with diverse antibiotics

Tobramycin conjugates have also been documented to synergize with several antibiotic classes, notably *β*-lactams and/or BL/BLI combinations (Idowu et al., 2019a, 2020; Idowu et al., 2020; Berry et al., 2021). These hybrid molecules consist of a secondary domain linked to tobramycin via an alkyl tether and lack ribosomal binding activity with the *C*-5 position used as a point of attachment. The structure–activity relationship (SAR) studies of these tobramycin conjugates have been reported, demonstrating the significance of the tether length and hydrophobicity, physicochemical properties of the secondary moiety, and number of cationic groups (Idowu et al., 2020). The various secondary motifs that have been conjugated to tobramycin include antibiotics [rifampicin (Idowu et al., 2019c), PMB_3_ (Domalaon et al., 2017) and fluoroquinolones (Gorityala et al., 2016a, 2016b; Dhiman et al., 2024)], antiparasitic agents [niclosamide (Berry et al., 2021)], EPIs (Yang et al., 2017) (NMP, paroxetine, and dibasic peptide analog of MC-04,124), chelating agents [cyclam (Idowu et al., 2019a), deferiprone (Gandhi et al., 2023)], and peptides (Lyu et al., 2017, 2019). Homodimeric analogs wherein tobramycin was conjugated to another tobramycin unit was also developed (Idowu et al., 2019b; Idowu et al., 2020). Despite the vast structural complexities between the secondary domains, they are mainly lipophilic or polybasic in nature and influence the amphiphilicity of the tobramycin conjugates with the accompanying aliphatic linker. Reducing the overall cationicity to mitigate toxicity was also accomplished by replacing the aminoglycoside scaffold with nebramine (Ammeter et al., 2019; Yang et al., 2019), the hydrolysis product of tobramycin which lacks the kanosamine sugar.

**Figure 5:**
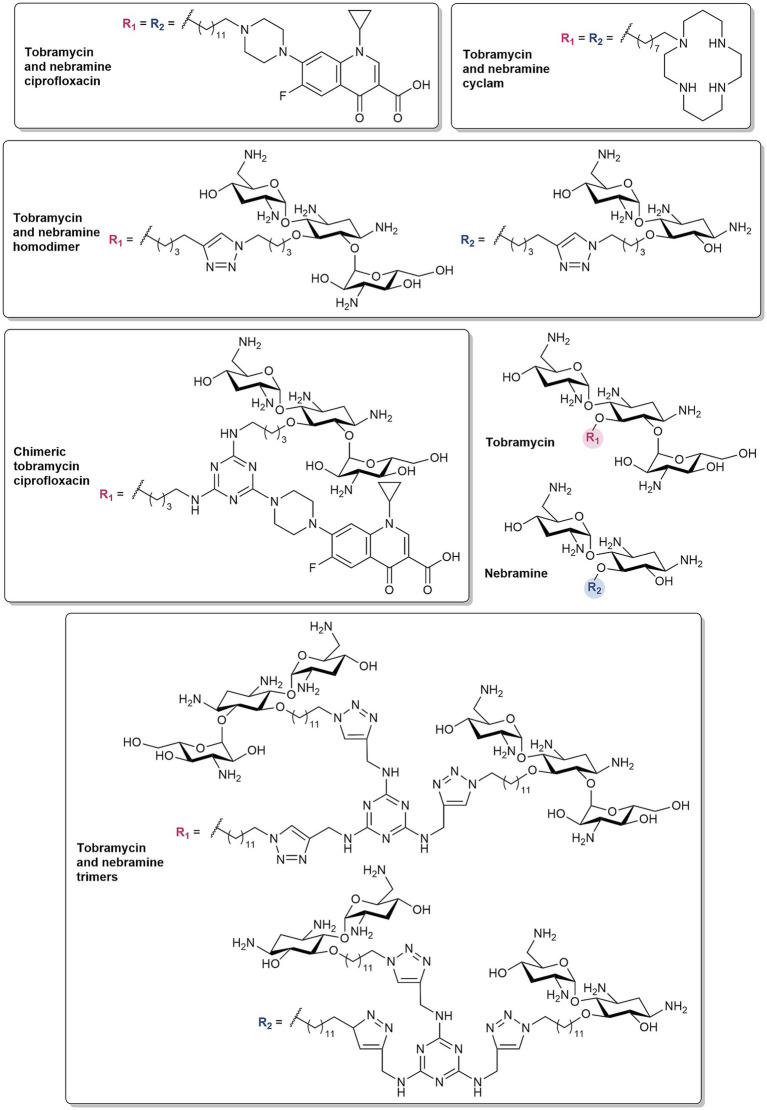
Structures of tobramycin and nebramine-based hybrids, homodimers, chimeric and trimeric molecules.

The most notable among these conjugates are the tobramycin-ciprofloxacin and -cyclam hybrids and the homodimeric tobramycin (Figure 5). The tobramycin hybrid conjugated to the piperazine of ciprofloxacin via a C12 chain (Figure 5) synergized with fluoroquinolones against MDR *P. aeruginosa* (Gorityala et al., 2016a), various *β*-lactams and BL/BLI combinations against β-lactamase-harboring *P. aeruginosa* (Idowu et al., 2020), as well with mitomycin C, an anticancer drug, against MDR Gram-negative bacteria (Domalaon et al., 2019). Further derivatives of this conjugate employed variation of the fluoroquinolone or aminoglycoside moiety (Gorityala et al., 2016b; Yang et al., 2019), conversion of the tobramycin amino groups to guanidines (Idowu et al., 2020), and modification of the linkers and point of attachment (Dhiman et al., 2024). The tobramycin- and nebramine-ciprofloxacin derivatives demonstrated comparable potentiation of rifampicin, fluoroquinolones, and minocycline against *P. aeruginosa* (Yang et al., 2019). Similarly, no significant differences in the potentiation of β-lactams and BL/BLIs against *P. aeruginosa* harboring β-lactamases were observed between the guanidinylated and non-guanidinylated tobramycin-ciprofloxacin hybrids (Idowu et al., 2020). In varying the flexibility and hydrophobic threshold of the tether, the 12-long carbon chain linker demonstrated enhanced tetracycline potentiation in comparison to the less hydrophobic polyethylene glycol and shorter C6 chain linkers, as well as the more rigid biphenyl tether (Dhiman et al., 2024). The tobramycin-cyclam hybrid with an eight-long carbon chain linker (Figure 5) enhanced in vitro activity and *in vivo* efficacy of β-lactam monotherapy and BL/BLI dual combinations against β-lactamase-harboring *P. aeruginosa* (Idowu et al., 2019a). Meanwhile, the homodimeric tobramycin, which comprises of a 1,4-dibutyl-1,2,3-triazole core joining the two tobramycin molecules, in combination with ceftolozane, was more active than ceftolozane/tazobactam against MDR or extensively drug-resistant (XDR) *P. aeruginosa* (Idowu et al., 2020). The tobramycin homodimer (Figure 5) was also found to be more potent than PMBN in potentiating novobiocin against MDR/XDR Gram-negative bacteria (Idowu et al., 2019b). Both tobramycin-cyclam and homodimeric tobramycin were comparable with their nebramine counterparts in potentiating *β*-lactams and BL/BLIs. The optimal linker length for these hybrids and homodimers were determined to be 8 or 12-carbon atoms long (Idowu et al., 2020).

A recent development to the tobramycin- and nebramine-based hybrids and homodimers, is the addition of a third domain (Dhiman et al., 2023b; Dhiman et al., 2023a; Figure 5). These structures consist of a central 1,3,5-triazine framework, appended with various combinations of tobramycin, ciprofloxacin, NMP, and cyclam to yield tobramycin-based chimeras (Dhiman et al., 2023b). The chimeric compound which contained 2 units of tobramycin and ciprofloxacin (Figure 5) synergized with fluoroquinolones and BL/BLI combinations against fluoroquinolone-resistant and *β*-lactamase-harboring *P. aeruginosa*, respectively (Dhiman et al., 2023b). In the chimeric series, compounds with two tobramycin moieties demonstrated higher antibiotic potentiation (Dhiman et al., 2023a). These results led to the incorporation of 3 units of tobramycin or nebramine with varying hydrocarbon chains to generate trimeric molecules (Dhiman et al., 2023a). The resulting tobramycin and nebramine trimers (Figure 5) were also synergistic with *β*-lactams and BL/BLI combinations against MDR *P. aeruginosa* (Dhiman et al., 2023a).

#### 2.2.2 Amphiphilic tobramycins enhance activity of multiple antibiotic classes

**Figure 6:**
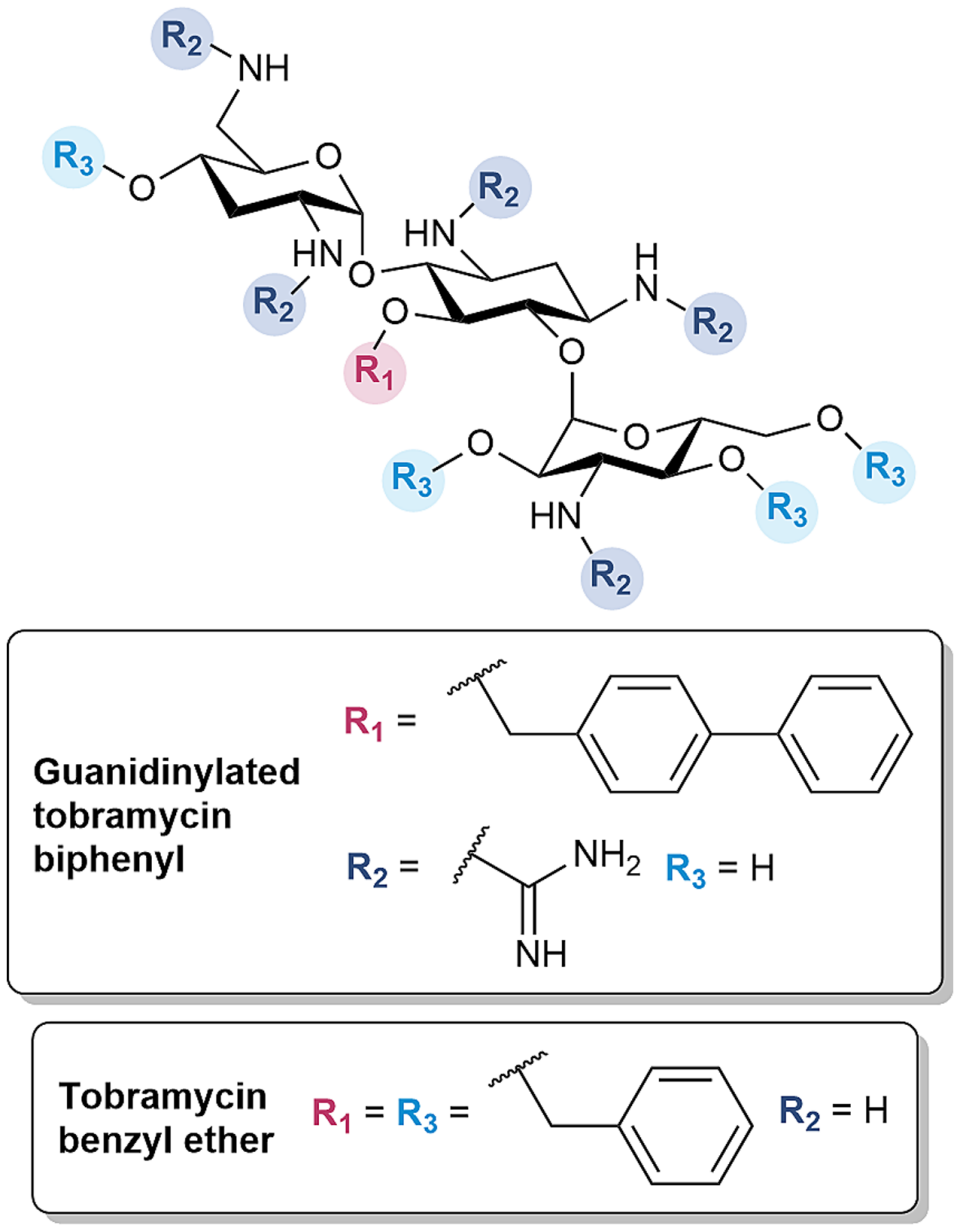
Structures of amphiphilic tobramycin derivatives.

Amphiphilic tobramycins were revisited for their capacity as OM permeabilizers, as prior derivatives were assessed solely for their immunomodulatory effects (Guchhait et al., 2015). Following established protocols (Guchhait et al., 2015; Idowu et al., 2019c) and SAR studies (Idowu et al., 2020), the amenable *C*-5 position was chosen for the installment of several lipophilic moieties to investigate their influence on OM permeabilization, given their critical role in facilitating the insertion within the lipid-rich region of the LPS. The derivatives were further optimized by converting the amines to guanidine functions (Figure 6), based on the same rationale for the development of guanidinylated polymyxins (Ramirez et al., 2020, 2022). The optimized guanidinylated tobramycin biphenyl derivative synergized with *β*-lactams, restoring the susceptibility of MDR and β-lactamase harboring *P. aeruginosa* to ceftazidime and aztreonam in both dual and triple combinations with avibactam, respectively (Ramirez et al., 2023). In particular, the triple combination of ceftazidime/avibactam plus guanidinylated tobramycin biphenyl resulted in a bactericidal effect (Ramirez et al., 2023). An indication of the OM permeabilizing capability of guanidinylated tobramycin biphenyl was substantiated by the time- and adjuvant concentration-dependent uptake of NPN, as well as the potentiation of rifampicin (Ramirez et al., 2023). More importantly, the derivative was non-cytotoxic against HEK293 cells up to 106 times the active concentration (Ramirez et al., 2023). The hit molecule also emphasized that the guanidinium and biphenyl groups were key structural features (Ramirez et al., 2023). Guanidinylation and/or alkylation resulted in a loss of antibacterial activity (Ramirez et al., 2023). These findings were consistent with previous studies, indicating the importance of the amino groups and *C*-5 hydroxyl in binding with the ribosomal RNA (Ramirez et al., 2023). However, the requirement of both components for synergism with *β*-lactam antibiotics was in contrast with previously synthesized tobramycin conjugates, wherein guanidinylation was not necessary and only showed comparable antibiotic potentiation. While the guanidinylated tobramycin derivatives were overall less cytotoxic compared to the polymyxins, guanidinylation still contributed to a slight increase in cytotoxicity (Ramirez et al., 2023).

The alkylation at the singular *C*-5 position in the aminoglycoside scaffold was further extended to include all other hydroxyl groups. Previously, Fridman et al. developed a synthetic strategy which allowed etherification of all alcohols in the tobramycin and nebramine scaffold (Berkov-Zrihen et al., 2015). Several of these cationic amphiphiles demonstrated promising low hemolytic activity and the ability to interact with LPS or disrupt the bacterial membrane (Berkov-Zrihen et al., 2015). Thus, these previous findings guided the design of benzyl and isopentyl tobramycin and nebramine ethers as OM permeabilizers. In addition, tobramycin phenyl carbamates were developed to ascertain the optimal point of attachment, since earlier studies only evaluated the standalone activity of these derivatives (Bera et al., 2010). Further structural optimizations included synthesis of substituted benzyl analogs to determine the necessary hydrophobic threshold required for OM permeabilization. The previously developed tobramycin benzyl ether (Figure 6) sensitized reference and resistant isolates of Gram-negative bacteria to rifampicin, and the restored susceptibility of drug-resistant *Escherichia coli* to minocycline (Ramirez et al., 2024). The OM perturbation of the compound was verified by its ability to mediate uptake of NPN, synergize with other OM impermeable antibiotics such as novobiocin and vancomycin, and lose synergistic interactions in the presence of competing Mg^2+^ and Na^+^ cations (Ramirez et al., 2024). While rifampicin potentiation did not translate to Gram-positive bacteria, tobramycin benzyl ether exhibited potent standalone activity against methicillin-resistant *Staphylococcus aureus* (MRSA) and vancomycin-resistant Enterococci (VRE) strains (Ramirez et al., 2024). However, the derivative showed non-specific binding to serum proteins, evidenced by the reduced rifampicin potentiation with the addition of fetal bovine serum (FBS) (Ramirez et al., 2024).

## 3 Limitations and future considerations

There are several challenges that should be considered in developing OM permeabilizers. Firstly, the inherent nephrotoxicity of both polymyxin- and aminoglycoside-based adjuvants must be mitigated. The polybasic and amphiphilic nature of these compounds which is required for OM permeabilization is also correlated to its toxicity. While aminoglycoside-induced nephrotoxicity can be managed by once daily dosing, polymyxins are still reserved as last-resort antibiotics. However, the development of the non-toxic polymyxin derivative SPR741 (Vaara et al., 2010) highlights that recent advancements have significantly improved the toxicity profile of this class of compounds. Secondly, in selecting the partner antibiotic, the base therapy needs to be effective. The OM perturbant may prevent emergence of resistance but it will not solve existing resistance. Furthermore, if both the OM permeabilizer and antibiotic lack standalone activity, it would be problematic from a regulatory perspective (Vaara, 2019). For these reasons, OM impermeable agents such as rifampicin may only be useful as model antibiotics, despite benefiting the most from enhanced OM permeability. Despite its safety and tolerability, rifampicin has limited activity against Gram-negative bacteria, and resistance occurs easily due to mutations in the *rpoB* gene (Vaara, 2019). Thus, it is an important consideration to choose an antibiotic partner that is effective as monotherapy. While *β*-lactams and/or BL/BLI combinations may be more suitable, it is imperative to take into account studies suggesting that the addition of an aminoglycoside to a *β*-lactam are related to higher rates of adverse effects, and may not be particularly beneficial in delaying or preventing resistance development, relative to β-lactam monotherapy (Bliziotis et al., 2005). Secondly, careful matching of the pharmacokinetic (PK) and pharmacodynamic (PD) properties of each individual component is required. For instance, aminoglycosides rely on maximizing concentration (C_max_/MIC) by single dosing (Krause et al., 2016), while β-lactams must be optimized for duration of exposure (T > MIC) which is increased by multiple dosing (Rybak, 2006). PK-PD predictions also demonstrate efficacy, however, it is difficult to apply these models to an agent which lacks a measurable MIC (Rex et al., 2019). The absence of direct antibacterial activity of either the adjuvant or antibiotic partner consequently necessitates compelling evidence (Rex et al., 2019). Lastly, even if the combination of the OM permeabilizer and antibiotic was demonstrated to be more effective than the current treatment, this superiority study is not usually used for new antibiotics. Instead, non-inferiority trials are used, wherein the new drug or combination must not be worse or have similar efficacy than the established therapy (Rex et al., 2019; Ofori et al., 2023). Therefore, it could be interpreted that utilizing an OM permeabilizer and an antibiotic does not provide any substantial benefit. While SPR741 was the most promising in this category of adjuvants, it is no longer in development, and the lack of other candidates in the current antibiotic pipeline emphasizes these difficult hurdles. Nonetheless, OM permeabilizers have the vast potential to allow uptake of a plethora of agents with novel targets that are restricted by the OM. If one were to be successfully approved in the future, it will only open doors to antibiotic discovery and a path to safeguard the future from the threat of AMR.

## 4 Conclusion

The development of polymyxin- and aminoglycoside-based OM permeabilizers leverages the unique ability of these antibiotics to interact with and disrupt the OM and represents a promising strategy to overcome the formidable permeability barrier of Gram-negative bacteria. This approach addresses the burden caused by AMR by revitalizing the use of existing antimicrobials, enhancing their efficacy or broadening their activity spectrum, as well as delaying resistance development. Continued optimization and mechanistic understanding of these agents will be key to their successful clinical translation.
